# Editorial: Performance Modeling and Anti-doping

**DOI:** 10.3389/fphys.2019.00169

**Published:** 2019-03-01

**Authors:** Raphael Faiss, Martial Saugy, Louis Passfield, James Hopker

**Affiliations:** ^1^REDs – Research & Expertise in Antidoping Sciences, University of Lausanne, Lausanne, Switzerland; ^2^School of Sport and Exercise Sciences, University of Kent, Chatham, United Kingdom; ^3^Faculty of Kinesiology, University of Calgary, Calgary, AB, Canada

**Keywords:** doping, sports, performance, modeling, biological passport

Medals shine under the spotlight for the winning athletes. In the context of global sport, athletic performance is scrutinized more than ever and the fight against doping is often considered as the shady side of the medal.

The Athlete's Biological Passport (ABP) was developed in an attempt to impede athletes' use of substances identical to those naturally produced by the human body (Sottas et al., 2011). Since its progressive implementation, the APB has become a strong tool for the indirect detection of doping (in blood) (Saugy et al., 2014; Zorzoli et al., 2014). Athletes aim to improve athletic performance via doping, but these practices may also influence biomarkers measured longitudinally as part of the ABP. However, numerous confounding factors (e.g., exercise training, hypoxic exposure, heat stress) are also known to alter these ABP parameters (Bouchard, 2015). There is therefore a need to gather additional information on athletes to strengthen the ABP, and provide a more forensic style intelligence led approach to anti-doping. One such approach is afford by the recent growth of technology in sports affording the ability to analyse and large volumes of data from both training and performance. Indeed, experts and scientists have gathered rudimentary performance data for decades to better understand the mechanisms underlying performance production (Faria et al., 2005; Borresen and Lambert, 2009; Sweeting et al., 2017), and with the aim of objectifying successes and failures of training strategies (Jobson et al., 2009; Passfield et al., 2017). The potential use of performance data for anti-doping purposes has only relatively recently been proposed (Schumacher and Pottgiesser, 2009), but has led to heightened interest in the area.

The objective of this Research Topic is to discuss the potential for scientific evidence-based models of athletic performance to provide a cost effective tool that can be used by anti-doping organizations in the fight against doping in sports. This research topic initially considers the outlook for athlete performance monitoring within an anti-doping context (and beyond!) from scientific experts of the anti-doping community (Iljukov and Schumacher; Hopker et al.). Next, it is interesting to consider how data can be utilized in the field to track changes in performance and adjust training strategies. In cycling for instance, power output is recorded extensively by nearly all professional teams and athletes both during training and races. This allows, for example, the use of peak power profiles to monitor training load and to adjust training programs to reach peak fitness at certain moments of the season (Pinot and Grappe, 2011, 2015). Since the ABP was first adopted in cycling by the Union Cycliste Internationale (UCI) in 2009 (by tracking hematological changes in professional cyclists), there has been a desire to better exploit this data by incorporating changes in performance in order to improve targeting. The development and selection of an adequate model is the first step that needs to be addressed, with some propositions in this research topic (Menaspà and Abbiss; Montagna and Hopker; Puchowicz et al.).

Concretely, Montagna and Hopker address the use of athlete performance data with a Bayesian approach much similar to the monitoring of hematological parameters in the ABP. Puchowicz et al. then propose a model specific to cycling using calculation of critical power (i.e., modelof the power-duration curve) to interpret performance variations. Such pragmatic approaches for cycling are put in perspective by Menaspà and Abbiss for the operational application in the ABP. Iljukov et al. additionally illustrate in practical ways how “unusual performances by an athlete would trigger a more thorough testing program” with a case report in middle- and long-distance runners. Moreover, Iljukov and Schumacher bring practical examples in 800 m runners, discus and hammer throwers with respective performance analyses. The latter shows the way to increase the efficiency of anti-doping measures by adjusting targeted testing using performance data.

There is also novel data on how cobalt may alter both hemoglobin mass and aerobic performance (Hoffmeister et al.), and an innovative statistical code tool allowing the calculation of the Abnormal Blood Profile Score marker as used in the ABP (Schütz and Zollinger). Finally, one should consider the opinion brought by a group of experts underlining the need for robust performance data before considering performance modeling (e.g., with the use of micro-technology monitoring activity and training) but also the potential of performance models in terms of risk prediction to identify athletes who are more likely to be involved in doping (Hopker et al.).

The body of evidence provided in this Research Topic supports the direction proposed in the ABP guidelines (Menaspà and Abbiss). This direction is that the term passport shall “include all other relevant information also comprising training and competition results” (Vernec, 2014). Thus the ABP could consist not only of a longitudinal profile of the athlete's hematological markers, but also considers performance models (including competition results and training contents) to formally support the ABP too. To date, more conclusive evidence highlighting associations between variations in the ABP and performance changes in competitive athletes is required. Defining links between existing or new biomarkers and performance would consequently represent an attractive strategy for indirect detection of the use of doping substances or methods. Moreover, the longitudinal monitoring of additional performance variables in different sports could be used to identify athletes “at risk” of doping worthy of closer scrutiny by anti-doping authorities. The aim of this Research Topic is ultimately to collect and discuss new evidence defining associations between performance models from various sports and existing or novel performance models to strengthen the fight against doping. Addressing this topic may help support anti-doping agencies seeking to remove the shady side of the medal when under the spotlight.

## Author Contributions

All authors listed have made a substantial, direct and intellectual contribution to the work, and approved it for publication.

### Conflict of Interest Statement

The authors declare that the research was conducted in the absence of any commercial or financial relationships that could be construed as a potential conflict of interest.
